# Lay Public View of Neuroscience and Science-Based Brain Health Recommendations in Slovenia

**DOI:** 10.3389/fpubh.2021.690421

**Published:** 2021-07-01

**Authors:** Nastja Tomat, Matej Perovnik, Gaj Vidmar, Vesna van Midden, Sara Fabjan, Hana Hawlina, Dolores Trol, Alina Holnthaner, Sebastijan Krajnc, Maruša Grešak, Liza Žerdin, Judita Vidmar, Mara Bresjanac

**Affiliations:** ^1^Institute for the Deaf and Hard of Hearing Ljubljana, Ljubljana, Slovenia; ^2^SiNAPSA, Slovenian Neuroscience Association, Ljubljana, Slovenia; ^3^Department of Neurology, University Medical Center, Ljubljana, Slovenia; ^4^Faculty of Medicine, University of Ljubljana, Ljubljana, Slovenia; ^5^University Rehabilitation Institute, Ljubljana, Slovenia; ^6^Faculty of Mathematics, Natural Sciences, and Information Technologies, University of Primorska, Koper, Slovenia; ^7^Department of Applied Kinesiology, Faculty of Health Sciences, University of Primorska, Koper, Slovenia; ^8^UMNI Institute, Ljubljana, Slovenia

**Keywords:** disease prevention, health literacy, public engagement, brain disorders, brain health

## Abstract

**Background:** Brain health is one of the cornerstones of a long and full life. Active care for brain health and reduction of lifestyle-related risks for brain disorders may be a key strategy in tackling the growing prevalence of mental and neurological illnesses. Public knowledge, perception, and preventive behavior need to be considered in the planning of effective strategies for brain health promotion. Our research is the first effort aimed at assessing Slovenian lay public knowledge, search and use of scientific information about the brain, and care for brain health.

**Methods:** An online survey was used to gather data for descriptive and associative statistical analyses of a sample of the Slovenian public (*n* = 2568) in August 2017. Participants with formal brain-related education were excluded, leaving the remaining sample of the lay public (*n* = 1012). Demographic characteristics and information regarding the perceived importance and knowledge of brain health and engagement in preventive behaviors of participants were collected, and key associative analyses were carried out.

**Results:** The majority of respondents (89%) considered brain health to be important. Over one-third (39%) considered their knowledge of the brain as sufficient relative to their needs. Most of the respondents identified science-recommended practices to be important for brain health. No recommendation was followed daily by the majority of the respondents, primarily due to declared lack of time (59%), and lack of information (32%). Information was obtained primarily from television (38%), followed by newspapers and magazines (31%), the Internet (31%), and direct conversations (27%). However, the highest-rated, preferred source of information was lectured by experts. One-third of our sample struggled with the trustworthiness of information sources. Female gender and older age were associated with a higher frequency of healthy practices. Personal or familial diagnoses of brain disorders were not associated with a higher frequency of the behavior in favor of brain health, but did affect available time and perceived value of preventive practices.

**Conclusions:** Our research provides an initial insight into the perceptions, knowledge, and brain health-promoting behavior of the Slovenian lay public. Our findings can inform future strategies for science communication, public education and engagement, and policy-making to improve lifelong active care for brain health.

## Introduction

Brain health is an emerging concept without a universally accepted definition (1). The World Health Organization defines brain health as a concept encompassing neural development, plasticity, functioning, and recovery across the life course (2). Individuals with good brain health experience optimal cognitive, emotional, psychological, and behavioral functioning enabling them to cope well with everyday challenges. According to American Heart Association/American Stroke Association (AHA/ASA), optimal brain health is an optimal capacity to function adaptively in the environment (3). Features of a healthy brain are the ability to pay attention, learn and remember, communicate, solve problems and make decisions, support movement, and regulate emotions (4). Various other definitions of brain health also include preservation of cognitive functions and the absence of neurological disorders (1, 5). Regardless of which definition one adopts, maintaining brain health is one of the cornerstones for a long and full life (1, 5). However, because brain disorders (BDs), manifested as neurological and mental illness, present a heavy global burden, and are a major cause of disability and death, long and full life is often not achieved (1, 6). In 2016, neurological disorders were the second leading cause of death and the leading cause of disability-adjusted life years [DALYs; calculated as the sum of years of life lost and years lived with disability (YLD)] globally (7). In the past 30 years, the number of deaths caused by neurological disorders has risen by 39% and DALYs have risen by 15% (7). Nearly one in three people in the world will be diagnosed with a neurological disorder in their lifetime (8). Neurological disorders that account for most DALYs are stroke, migraine, and Alzheimer's disease and other dementias (7). In 2010, mental and substance use disorders alone were the leading cause of non-fatal burden of disease, calculated as YLD, and the fifth largest contributor to DALYs worldwide (6). In the same year, the total cost of BDs in Europe was estimated to be 798 billion €, positioning BDs as the number one public health challenge (9). In addition to great financial costs, BDs represent a big psychosocial burden for patients, their caregivers, and society in general (10–13). It is predicted that, due to population aging, the prevalence of brain disorders will continue to rise, which is of great concern for health systems around the world (7). Based on the methodology used by the European Brain Council study (14), estimates of BDs costs for Slovenia in 2010 showed similar results. Financial burden of BDs was estimated at 7% of gross domestic product and approximately one-third of all health care costs in Slovenia (9). As Slovenia's population is aging rapidly (15), the prevalence of BDs is expected to rise (16).

The goal of public health programs is disease prevention, life prolongation, and promotion of health and well-being *via* different approaches, including promotion of healthy behavior and reduction or alteration of risk factors for disease (17, 18). Despite extensive research, many aspects of brain functioning in health and disease are not yet understood (19). Mechanisms and causes of many BDs are still unknown and there is a lack of established risk factors that contribute to their development (8, 17). Nevertheless, there are several evidence-based recommendations for maintaining brain health, targeting the general population at the primary prevention level. Many risks are common to both non-communicable medical disorders and common neurological and mental disorders (e.g., poor diet, smoking, and physical inactivity) and lend to universal strategies with known and empirically validated efficaciousness, which allows compound benefits for singular interventions (20). For example, AHA/ASA recommends the management of blood pressure, controlling cholesterol, reducing blood sugar, physical and social activity, a healthy diet, cessation of smoking and limiting alcohol intake, weight management, and sufficient restful sleep for maintaining brain health (4).

Public education and engagement in sustaining lifelong health are one of the key tasks in many national resolutions and public health strategies (21, 22). However, brain health promotion and prevention of BDs have received far less attention and engagement than prevention of other large non-communicable disease groups, such as cancer and cardiovascular disorders (23), which may in part be due to the perceived complexity of brain-related topics. Insight into the current state of public understanding and use of scientific knowledge about the brain is a prerequisite for developing strategies to engage citizens in active care for their own health and primary prevention of BDs (24). Such research may inform strategies, approaches, and engagement activities for communication and policy-making for lifelong brain health and may in the long term reduce the burden of BDs. The goal of our study was to investigate the Slovenian lay public's knowledge, search and use of scientific information about the brain, brain research, and care for brain health. We were also interested in identifying possible obstacles to public access and adherence to science-based recommendations for brain health. To gather relevant data, we designed and carried out an online survey. This was the first such study in Slovenia.

## Methods

### Procedure

This was a cross-sectional observational study in the form of a survey. The data were collected within the project “Z možgani za možgane” (Aim for the Brain) in August 2017. The survey participants were asked to: (1) rate their knowledge about the brain and perceived importance of brain health (using a five-point scale); (2) state their perceived importance of, and engagement in, activities that promote brain health (choosing from a list of options); and (3) report on their experience with brain-related information sources (selecting from a list of options). In some instances, participants were able to enter comments and provide their own answers. Basic demographic data were also collected. The survey was published on two different online platforms, 1 ka (University of Ljubljana, Ljubljana, Slovenia), and Qualtrics (Qualtrics, Provo, UT, United States). The whole survey (in Slovenia) can be reviewed at https://www.1ka.si/a/280638. As opposed to the original survey, the version included here was adjusted so that responses to the questions are not mandatory.

### Participants

Participants were recruited *via* social platforms (e.g., the project's Facebook page), mailing lists (e.g., the Slovenian neuroscience association (SiNAPSA) and various student organizations), and partner websites (http://umni.si/). Participation was conditioned on candidate familiarization with the aim and scope of the survey, and declaration of their informed consent to the survey requirements. Besides fluency in Slovene, there were no specific criteria for the inclusion of participants. At the launch of the survey, it was announced that a number of practical health-friendly prizes will be awarded to participants selected by a random draw. The links to the survey were active for 3 weeks.

### Data Analysis

We calculated descriptive statistics and presented the distributions of the responses graphically. Some questions and answers are abridged in the results; for the complete survey see Supplementary Material. The difference in mean rank between preferred information sources was statistically assessed using Friedman's test. In addition, we executed exploratory analyses of associations between survey responses and respondents' characteristics. We investigated whether the participants' age, gender, and the presence of a personal or familial diagnosis of a brain disorder were correlated with the preferred way of obtaining new information, adherence to prevention practices, and obstacles to engagement in those practices. The association between groups and preferred source of information about the brain was assessed by using the Chi-squared test (with Monte-Carlo *p*-value based on 10,000 samples). The difference in the number of preventive practices performed by men and women was assessed with an independent *t*-test. The difference in the number of preventive practices performed regarding age group and the neurological or psychiatric diagnosis was assessed with a one-way analysis of variance (ANOVA). IBM SPSS Statistics 23 (IBM Corp. Armonk, NY, United States) software was used for data analyses.

## Results

**Figure 1:**
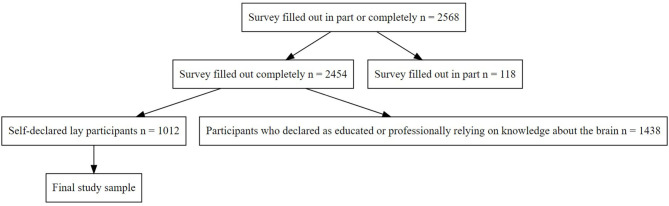
Flowchart of the selection procedure.

The survey was filled out in part or completely by 2,568 participants. The final sample included 1,012 participants; the selection process is shown in Figure 1. All data and analyses reported here pertain only to this subsample. The demographic characteristics of the lay participants are shown in Table 1.

**Table 1:** Demographic characteristics of lay participants.

**Variable**	***n***	**%**
**Gender**
Female	709	70.1
Male	300	29.6
Other	3	0.3
**Age ^a^ (years)**
≤ 19	83	8.3
20–27	289	28.9
28–50	452	45.2
≥51	184	18.4
**Education**
Secondary school/less	321	31.7
Bachelor's degree	421	41.6
Master's/doctoral degree	270	26.4
**Region**
Central Slovenia	478	47.2
Podravje	137	13.5
Gorenjska	109	10.8
Savinjska	64	6.3
Other	224	22.2
**Employment status ^b^**
Employed	637	59.7
Student	290	27.2
Retired	50	4.7
Unemployed	63	5.9
Inactive	27	2.5

a *Four missing values*.

b *Multiple answers were possible*.

### Descriptive Statistics

Most (89%) of the respondents (*n* = 999) deemed brain health as important (37%) or as one of the most important things in life (52%). Only 0.2% responded that brain health is not important at all.

Most participants (*n* = 999) described themselves as either having some knowledge of the brain (45%) or rated their knowledge of the brain as sufficient (39%) relative to their needs; 8% responded that they have no or almost no knowledge of the brain, 7% rated their knowledge as good enough to cover their needs, and 1% rated their knowledge excellent.

**Figure 2:**
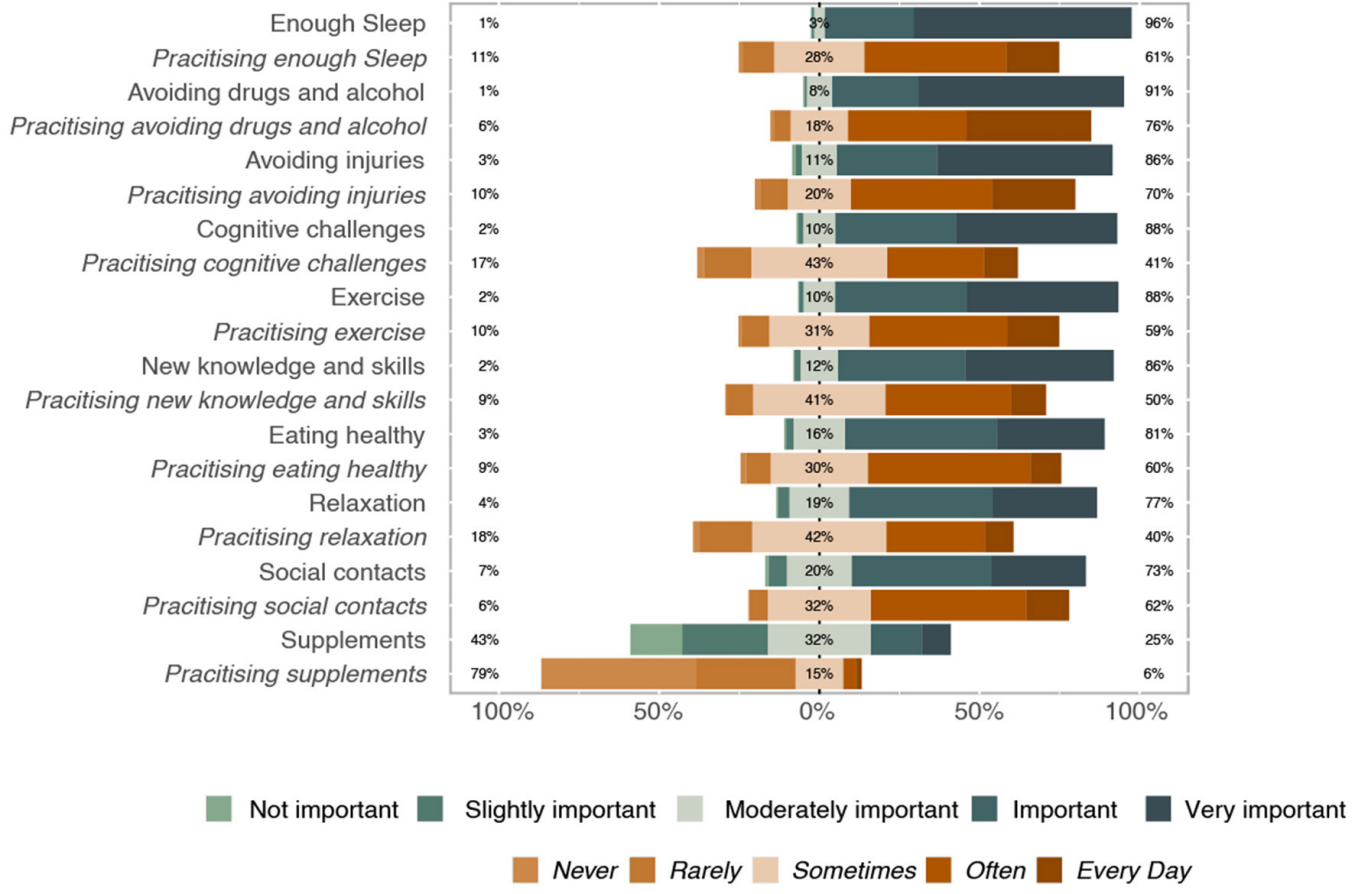
Percentages of responses to the questions “How do you evaluate the following practices in terms of their value for brain health?” (*n* = 945, color-coded green to blue) and “How frequently do you comply with recommendations for brain health?” (*n* = 945, color-coded orange to brown).

**Figure 3:**
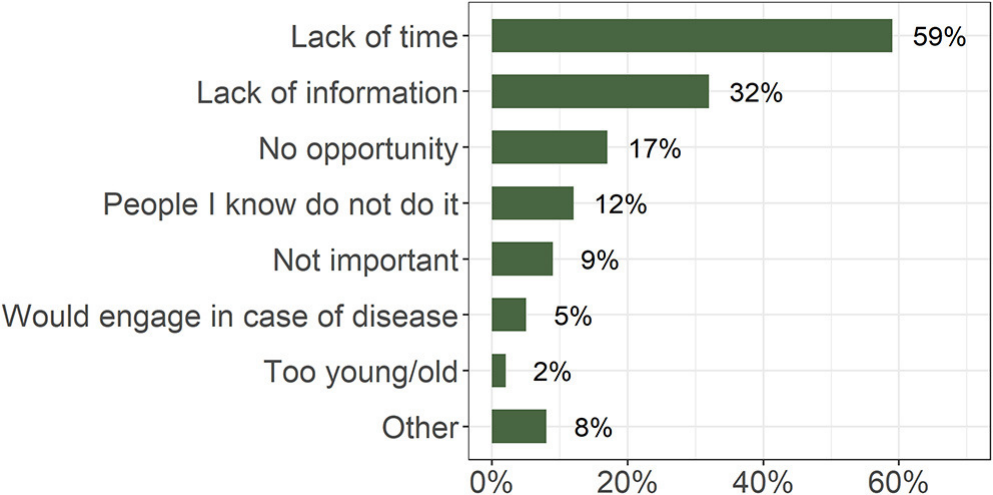
Percentage of responses to the question “Why don't you engage in healthy practices as much as you know would be good for you?” (*n* = 945; multiple answers possible).

More than 70% considered all practices listed in the survey, except the use of supplements for cognitive enhancement, to be very important for maintaining brain health (Figure 2). Of those options, sufficient sleep, avoiding drugs and alcohol, avoiding injury, and engaging in cognitive challenges were perceived as very important by most of the respondents. Reported behavior exhibited a similar pattern (Figure 2). No recommendation was followed daily by the majority of the participants. Lack of time and lack of information were the most commonly stated reasons for participants not engaging in the activities (Figure 3).

**Figure 4:**
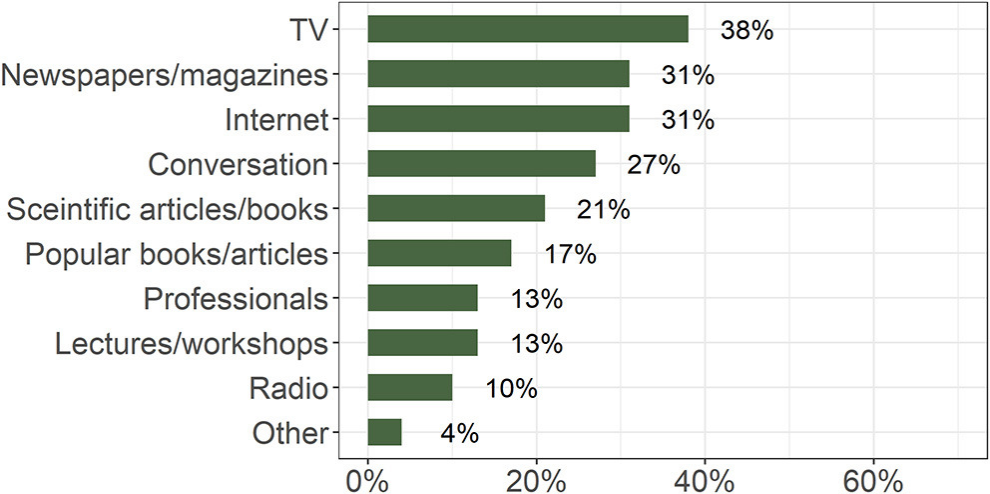
Percentage of responses to the question “Among the following sources choose the first three that you rely most on to gain information about the brain” (*n* = 1012).

Our participants stated that they most often obtained brain-related information from TV programs (38%), followed by newspapers or magazines (31%), and the Internet (31%) (Figure 4).

**Figure 5:**
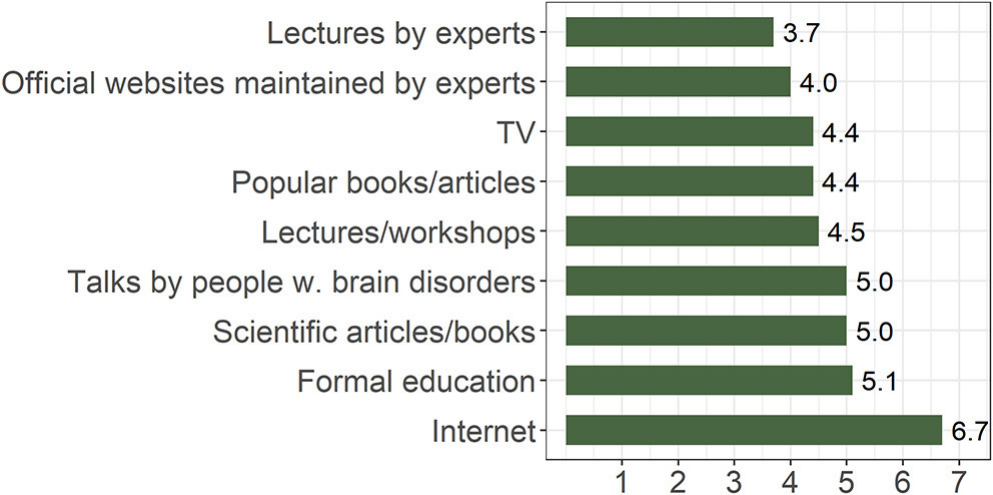
Median ranks of answers to the question “What is your preferred manner of acquiring new knowledge about the brain, brain disease, and preventive practices?” (lower rank indicates higher preference; *n* = 894).

Regarding the preferred manner of acquiring new knowledge about the brain, lectures by experts were ranked the highest on average, while the Internet (in general, not a particular site) was the least preferred (Figure 5). The difference in mean rank was statistically significant (χ2 (8) = 758.2, *p* < 0.001).

**Figure 6:**
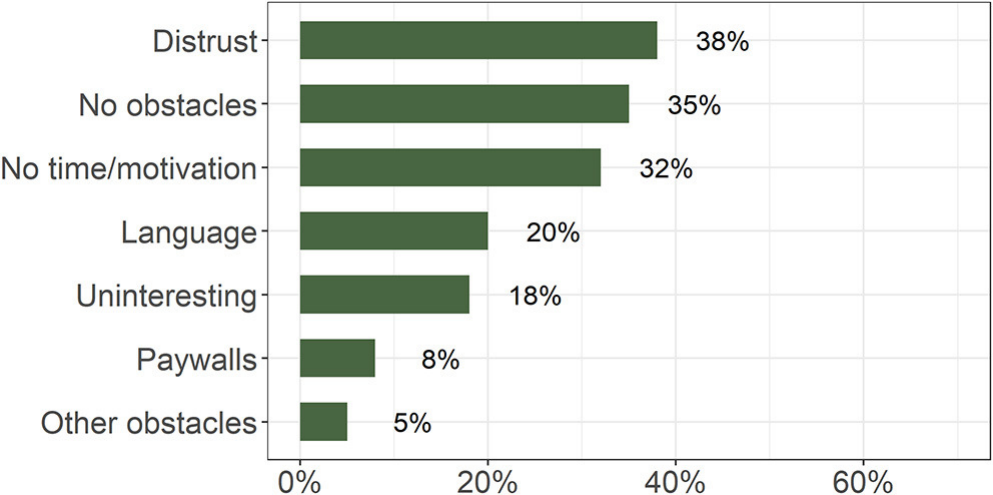
Percentage of responses to the question “Have you encountered any obstacles in searching for information about the brain?” (*n* = 887; multiple answers possible).

Although, approximately one-third of the participants (35%) reported that they did not encounter any obstacles when searching for information about the brain (Figure 6), a larger proportion (38%) stated that they struggled with the trustworthiness of sources.

### Associative Statistics

#### Preferred Source of Information

There were statistically significant differences between gender (χ*2* (8) = 19.071, *p* = 0.013; *V* = 0.15) and age groups *(*χ*2* (24) = 42.799, *p* = 0.010; *V* = 0.13) regarding the preferred source of information about the brain. No such difference was observed with respect to the presence or absence of personal or familial diagnosis of brain disease (χ2 (24) = 21.572, *p* = 0.606). More women than men (10 vs. 5%, respectively) chose learning from talks by people with BD as a preferred source of information, whereas, more men chose the Internet (8 vs. 5%, respectively) and from popular books and articles (12 vs. 8%, respectively). Among the youngest age group (19 years or less), the proportion of those preferring formal education about the brain was the highest (25 vs. 14% or less among the other age groups) and the proportion of those preferring official expert websites was the lowest (12 vs. 24% or more among other age groups); the proportion of those preferring popular books and articles was the highest in the oldest age group (51 years or more; 15 vs. 9% or less among other age groups).

#### Prevention Practices

On average, there was a statistically significant difference in the number of preventive practices performed by women compared with men (5.1 vs. 4.5, respectively; *t* (1007) = 3.432, *p* = 0.001; *g* = 0.24). The average number of performed practices increased with age (3.9 among those 19 years or younger, 4.7 and 4.9 among those 20–27 years and 28–50 years of age, respectively, and 5.7 among those 51 years or older; *F*_(3, 1004)_ = 13.571, *p* < 0.001; η*2* = 0.04). The average number of practices performed did not differ statistically significantly with respect to neurological or psychiatric diagnosis [*F*_(3, 1008)_ = 2.027, *p* = 0.108].

#### Obstacles to Engagement in Activities Conducive to Brain Health

Due to the nature of our survey (multiple possible responses), a simple statistical analysis of obstacles preventing respondents from engaging in activities conducive to brain health was not possible. However, we observed several differences when comparing answers based on gender, age, and the presence of a brain disorder diagnosis.

Women reported no opportunity as the obstacle more frequently than men (20 vs. 12%, respectively), whereas, more men than women deemed such engagement unimportant (13 vs. 7%, respectively).

The oldest age group (51 years or more) differed from younger groups in that more of its members reported engaging in all such activities (47 vs. 26% or less, respectively). Furthermore, compared with other age groups fewer participants from the oldest age group reported lack of time (40 vs. 65% or more), lack of information (27 vs. 32% or more), and no opportunity as an obstacle (10 vs. 16% or more). Conversely, members of the youngest age group (19 years or less) reported their age as a limitation more frequently (12 vs. 3% or less in the older groups).

Individuals with a diagnosis of a BD reported lack of time as an obstacle to engagement in activities conducive to brain health less frequently (49 vs. 60% or more among other age groups). However, a larger proportion of this group deemed such endeavors unimportant (16 vs. 9% or less among others). Individuals with family members diagnosed with a BD in addition to having such diagnosis themselves reported no opportunity more frequently than the others (30 vs. 19% or less among others).

## Discussion

The majority of the Slovenian lay public considered brain health to be important, but only 8% believed their knowledge was excellent or good enough to cover their needs. More than 70% considered practices, such as a healthy diet, exercise, and relaxation to be important for maintaining brain health. Lack of time and lack of credible science-based information were the two most frequently reported reasons for the general public not acquiring more knowledge about the brain and for not engaging in brain-healthy activities. Information supplied directly by experts was the preferred manner of knowledge acquisition. Questionable trustworthiness of sources was the most commonly stated obstacle in gathering information about the brain. Female gender and older age, but not a personal or familial diagnosis of brain disorders, were associated with a higher frequency of preventive practices.

In view of the crisis brought about by the epidemic of BDs both globally (14) and locally (9), it is of note that the vast majority of participants stated that they viewed brain health as important or one of the most important things in life. This may facilitate new strategies for improved science communication and public engagement. New approaches seem warranted, as the majority of our respondents stated that they had only some or almost no knowledge of the brain.

In congruence with scientific evidence, the majority of respondents correctly rated the beneficial effects of adequate sleep and avoidance of drugs and alcohol as important or very important for brain health (25–27). In contrast with numerous false advertisement campaigns (28, 29), cognition enhancing supplements were rated as the least important for brain health. Although, sufficient relaxation and social interactions were rated as important or very important by the majority of respondents, the perceived importance of these two activities was low in comparison to others. This may be because relaxation and maintaining social contacts may be viewed as having more psychological benefits than being healthy in a physical sense, possibly due to public perception of mental and physical health as separate entities (30). There is extensive evidence on the bilateral influences of mental and physical health (31–33) and this connection between brain health and psychological variables, e.g., overall well-being, should be accentuated, and its mechanisms explained to the public.

For the sake of brevity and focus, our study did not address if there are differences between the perceived importance of activities for maintaining general health vs. brain health. Since healthy nutrition and exercise are extensively promoted as beneficial for general health (34–38), it may be relatively straightforward to raise awareness of the connection between cardiovascular and metabolic health and a healthy brain, thus adding strength to the arguments in favor of a healthy lifestyle. Regrettably, the current Slovene resolution on health care for 2016–2025 (21) and the resolution on the national program for healthy nutrition and physical activity 2015–2025 (35) missed the opportunity to build on that argument. Both documents list only stroke and traumatic brain injury among chronic non-communicable nervous system disorders that may be ameliorated or prevented by a healthy lifestyle. Although, an important step was recently made toward tackling mental health challenges (39), there is still ample room and urgent need for strategies to improve all aspects of active care for brain health in Slovenia (9).

The predominant sources of information about the brain for our respondents were television, newspapers/magazines, and the Internet. They relied least on expert lectures or thematic workshops. Interestingly, expert lectures and expert-run websites were listed as the most desired means for acquiring information, indicating a communication gap that calls upon scientists and experts to fill. The Internet in general, not a particular site, was rated as the least-valued source. Together these findings point to the public interest in getting information directly from the experts and to the concurrent shortage of such direct communication.

Importantly, one frequently reported obstacle encountered by lay public when seeking information about the brain was distrust of the information sources. Considering the fact that vast amounts of unverified, often biased, and agenda-driven information are readily accessible and forced onto the public (40, 41), it is encouraging to find almost 40% of our respondents questioned the trustworthiness of the available information sources. Together with the expressed interest of the same sample of the lay public to obtain well-presented information directly from the experts, this indicates an opportunity for the Slovenian scientists, clinicians, and other professionals to fill the information void with understandable science-based brain health–promoting information tailored to various target audiences. Furthermore, critical appraisal of the available information in the media should be encouraged to further enhance public ability to weed out biased, non-scientific information outlets. Paywalls were also listed among obstacles, but we do not know if this relates to scientific publications or restricted social group information. With regard to the former, it is of note that open access publishing is gaining in volume and widely promoted (42, 43). Slovenian scientists and health care professionals seem to have ample room for improvement of our communication and public engagement skills.

Although the perceived importance of activities was high, and many healthy practices were rated as such, the majority of participants stated that they did not take care of their health on a daily basis. They reported the highest adherence to avoid drugs, alcohol, and brain injury, and maintaining social contacts, followed by getting enough sleep, and eating healthy. The discrepancy between the perceived importance of activities and the level of engagement may be due to the fact that change of behavior is often difficult even though we are aware of the benefits of such a change (44–46).

Although the majority of participants did not consider maintaining social contacts beneficial for brain health, more than 60% regularly engaged in it. Promoting the connection between rich social life and brain health can be used to further build on these synergies, e.g., using a buddy system or a team approach to foster physical activity and healthy nutrition (47).

The most commonly stated obstacles to adopting brain-healthy habits were a lack of time and a lack of actionable information. If people were to dedicate a larger proportion of their daily time to activities they perceive as important, the currently least practiced healthy activities have to either become subjectively more important or – when possible – more feasible. The former requires a shift in collective mentality and a coordinated action in the development of successful health promotion strategies adjusted to specific target groups (48–51). The lack of time calls for time-efficient ways to include healthy habits in daily routines. This could be accomplished by wider use of the available technology, e.g., smartphone applications that prompt users to engage in healthy activities (52, 53), promoting small health- and ecologically friendly changes in daily routine (e.g., taking the stairs or cycling to work) and stimulating local, organized exercise forms. The latter have already been shown to help increase the level of sports activities in Slovenia (54).

Preferred source of information differed in regard to gender and age. More men than women preferred gaining information on the Internet, from popular books and articles. On the other hand, more women than men preferred talks by people with brain disorders. Gender differences could be explored further and preferences of both genders should be taken into account when developing public education strategies.

The younger subset of our respondents listed formal education as the preferred way of gaining information in the highest proportion among all age groups. Surprisingly, the proportion of those preferring official websites maintained by experts was the lowest in this group which suggests that we should put more emphasis on brain health during formal education to effectively empower young people with knowledge and motivation for lifelong healthy practices. This is underscored by research findings, which show that behaviors adopted in childhood and adolescence tend to continue into adulthood and have an impact on health later in life (55–58).

On average, women were engaged in more preventive practices for brain health than men. This is in concordance with a large Chinese study, where authors observed a higher adherence to a healthy lifestyle in women compared with men (59). The average frequency of healthy practices also continuously increased with age; each older age group engaged in more activities than the previous one, with participants aged 51 years or more engaging in the most number of activities. Interestingly, personal and family diagnoses of brain disease did not influence the average frequency of healthy activities.

Women reported that they had no opportunity to engage in healthy activities more frequently than men, but men more often considered engagement in such activities unimportant, which likely explains lower engagement in healthy activities. More men than women also reported no obstacles when seeking information about the brain.

The differences in encountered obstacles to active engagement also emerged between age groups. Obstacles such as lack of time, lack of information, and no opportunity were less frequent in the oldest age group (51 years or more) compared with the younger groups. Members of the youngest age group (19 years and less) reported being too young as an obstacle far more frequently than other groups. This misconception should be sensibly targeted to improve young people's understanding of the role of early adoption of a healthy lifestyle in the prevention of brain diseases, as shown by an abundance of evidence (55–58). It is important to communicate the fact that most brain disorders are not a consequence of aging *per-se*, and even when their onset occurs later in life, their clinical presentation and course can be significantly modified by maintaining a lifelong commitment to a healthy lifestyle. An illustrative example is a head trauma, a leading cause of death in youth and a cause of chronic disability, which could be effectively prevented in the majority of cases by avoiding dangerous behavior and using protective gear (60). The second main cause of death among 15- to 29-year olds globally is a suicide, so awareness of mental health protection from an early age is of paramount importance (61–63).

The proportion of participants with a diagnosis of a BD who perceived preventive practices as unimportant was higher than that of the unaffected respondents (16 vs. 9%, respectively). This could imply that the illness reduced their perception of the benefits of prevention or that they deemed preventive practices unimportant even before they fell ill. Either way, these respondents were less motivated to actively participate in brain health–promoting activities and should be selectively targeted in any attempt to improve public engagement. Respondents with a familial diagnosis of a brain disorder reported no opportunity to engage in healthy activities more frequently than participants without the diagnosis. Caregiving relatives are at a higher risk of developing burnout syndrome, which by itself can also cause a severe reduction in the quality of life and leave permanent damage (13, 64).

Primary prevention can prevent or delay a proportion of BDs (65–67). A good example is dementia where a life-course model that was recently published by the Lancet Commission on dementia prevention, intervention, and care recognizes 12 modifiable risk factors that account for 40% of worldwide dementia, which could be prevented or delayed (65). Even if their primary disease cannot be prevented with a healthy lifestyle, such patients are still at risk for developing additional BDs and secondary conditions, such as obesity, diabetes, and hypertension that worsen chronic brain dysfunction. Such secondary conditions further diminish the patients' functional independence and quality of life (68).

Our study was the first of its kind in Slovenia, but our literature search revealed a paucity of similar explorations on an international scale (69–71). A significant and encouraging exception was recently provided by the Global Brain Health Survey by LifeBrain consortium (72) as it reaffirmed the value of systematic efforts to assess the perceptions of people on brain health and factors influencing brain health.

Future studies could further investigate how demographic characteristics of the participants (e.g., age, gender, education) are related to the attitudes and preferences regarding engagement in brain-healthy practices and knowledge acquisition. Targeting specific population, not only the lay public in general, could help develop more effective strategies for public education about brain health and engagement of citizens in active care for their own health and primary prevention of brain disorders.

Furthermore, it would be worthwhile to investigate how the lay public perceives the relationship between physical and mental health – more specifically, how they perceive the connection between brain health and activities that are typically promoted as beneficial for psychological rather than physical well-being (e.g., relaxation and maintaining social contacts). Based on that information, strategies for public education about the reciprocity of mental and physical well-being could be devised. Additional questions that warrant answers are what is the difference between the perceived importance of activities for maintaining general vs. brain health, and whether prevention could be improved by emphasizing the connections between them.

### Strengths and Limitations of the Study

The study includes a broad variety of topics: perceived importance of brain health in general, perceived importance of and adherence to health-promoting practices, and obtaining information about brain health. As such it serves as a good starting point for future studies that would target only selected groups and focus on more specific research questions. Exploratory analyses of associations between demographic characteristics of the participants and their answers provide an initial insight into preferences of specific subsamples that could be further investigated and targeted with specific strategies.

The limitation of the study was that in the Central Slovenian region, women, higher educated people, and students were overrepresented in our sample relative to the population at large (73–76). This was partly due to the method of data collection (an online survey), which limited our respondents to people with Internet access and basic computer skills, so certain groups (e.g., the elderly, underprivileged) were less likely to participate. Future studies could utilize a paper-and-pencil version of surveys in addition to digital ones and specifically target underrepresented groups (e.g., unemployed), as we believe this would provide valuable information required for developing effective targeted strategies in accordance with their needs. To improve their physical and mental health, the primary causes of marginalization and vulnerability of these groups should be addressed as a priority.

### Conclusions

We report the results of the first study of the general Slovene public interest in, attitude toward, and use of scientific findings of the brain, brain research, and maintenance of brain health. Lack of credible, understandable, science-based information in Slovenia was found to be one of the two most important reasons for the general public not acquiring more knowledge about the brain, and for not engaging in brain health–promoting activities. Information supplied directly by experts was the foremost desired manner of knowledge acquisition. Our findings can inform educational, science-promoting, and brain health–oriented strategies, and also serve as a baseline in designing of any future brain health–oriented strategies in Slovenia.

## Data Availability Statement

The raw data supporting the conclusions of this article will be made available by the authors, without undue reservation.

## Ethics Statement

Ethical review and approval was not required for the study on human participants in accordance with the local legislation and institutional requirements. Written informed consent from the participants' legal guardian/next of kin was not required to participate in this study in accordance with the national legislation and the institutional requirements.

## Author Contributions

NT, MP, and MB wrote and edited the manuscript. NT, MP, VM, SF, DT, AH, SK, MG, HH, and LŽ participated in study design and data collection. GV performed statistical analyses. MP prepared the figures. JV and MB supervised the team and contributed to conceptual framework and coordination. All authors contributed to manuscript revision and approved the final version.

## Conflict of Interest

The authors declare that the research was conducted in the absence of any commercial or financial relationships that could be construed as a potential conflict of interest.

## References

[1] Wang Y Pan Y Li H. What is brain health and why is it important? BMJ. (2020) 371:m3683. 10.1136/bmj.m3683 33037002 PMC7555053

[2] World Health Organisation. Brain Health. (2020). Available online at: https://www.who.int/health-topics/brain-health#tab=tab_1 (accessed January 13, 2021).

[3] Gorelick PB Furie KL Iadecola C Smith EE Waddy SP Lloyd-Jones DM. Defining optimal brain health in adults: a presidential advisory from the American heart association/American stroke association. Stroke. (2017) 48:e284–303. 10.1161/STR.0000000000000148 28883125 PMC5654545

[4] AHA/ASA. Control Stroke Risk Factors for Brain Health. Available online at: https://www.stroke.org/en/about-the-american-stroke-association/american-stroke-month/community-resources-english/control-stroke-risk-factors-for-brain-health (accessed January 13, 2021).

[5] Alzheimer's Association Center for disease control and prevention. Healthy Brain Initiative, State and local Public Health Partnerships to Address Dementia: the 2018-2023 Road Map. Chicago, IL. (2018). Available online at: https://www.cdc.gov/aging/pdf/2018-2023-Road-Map-508.pdf (accessed April 2, 2021).

[6] Whiteford HA Degenhardt L Rehm J Baxter AJ Ferrari AJ Erskine HE. Global burden of disease attributable to mental and substance use disorders: findings from the Global Burden of Disease Study 2010. Lancet. (2013) 382:1575–86. 10.1016/S0140-6736(13)61611-6 23993280

[7] Feigin VL Nichols E Alam T Bannick MS Beghi E Blake N. Global, regional, and national burden of neurological disorders, 1990–2016: a systematic analysis for the Global burden of disease study 2016. Lancet Neurol. (2019) 18:459–80. 10.1016/S1474-4422(18)30499-X 30879893 PMC6459001

[8] Feigin VL Vos T Nichols E Owolabi MO Carroll WM Dichgans M. The global burden of neurological disorders: translating evidence into policy. Lancet Neurol. (2020) 19:255–65. 10.1016/S1474-4422(19)30411-9 31813850 PMC9945815

[9] Bon J Koritnik B Bresjanac M Repovš G Pregelj P Dobnik B. Cost of disorders of the brain in slovenia in 2010 | Stroški možganskih bolezni v sloveniji v letu 2010. Zdr Vestn. (2013) 82:164–75. Available online at: https://vestnik.szd.si/index.php/ZdravVest/article/view/724 (accessed June 16, 2021).

[10] Vitaliano PP Zhang J Scanlan JM. Is caregiving hazardous to one's physical health? A meta-analysis. Psychol Bull. (2003) 129:946–72. 10.1037/0033-2909.129.6.946 14599289

[11] Wittchen HU Jacobi F Rehm J Gustavsson A Svensson M Jönsson B. The size and burden of mental disorders and other disorders of the brain in Europe 2010. Eur Neuropsychopharmacol. (2011) 21:655–79. 10.1016/j.euroneuro.2011.07.018 21896369

[12] Corrigan PW Watson AC Miller FE. Blame, shame, and contamination: the impact of mental illness and drug dependence stigma on family members. J Fam Psychol. (2006) 20:239–46. 10.1037/0893-3200.20.2.239 16756399

[13] Tramonti F Bonfiglio L Bongioanni P Belviso C Fanciullacci C Rossi B. Caregiver burden and family functioning in different neurological diseases. Psychol Health Med. (2018) 24:27–34. 10.1080/13548506.2018.1510131 30141703

[14] Gustavsson A Svensson M Jacobi F Allgulander C Alonso J Beghi E. Cost of disorders of the brain in Europe 2010. Eur Neuropsychopharmacol. (2011) 21:718–79. 10.1016/j.euroneuro.2011.08.008 21924589

[15] Statistical Office of the Republic of Slovenia. Population by Age and Sex, Municipalities, Slovenia. Available online at: https://pxweb.stat.si/SiStatData/pxweb/sl/Data/-/05C4002S.px/ (accessed January 31, 2021).

[16] Carroll WM. The global burden of neurological disorders. Lancet Neurol. (2019) 18:418–9. 10.1016/S1474-4422(19)30029-8 30879892

[17] Stanaway JD Afshin A Gakidou E Lim SS Abate D Abate KH. Global, regional, and national comparative risk assessment of 84 behavioural, environmental and occupational, and metabolic risks or clusters of risks for 195 countries and territories, 1990–2017: a systematic analysis for the global burden of disease study 2017. Lancet. (2018) 392:1923–94. 10.1016/S0140-6736(18)32225-6 30496105 PMC6227755

[18] World Health Organisation. Public Health Principles and Neurological Disorders. (2006). Available online at: https://www.who.int/mental_health/neurology/chapter1_neuro_disorders_public_h_challenges.pdf (accessed June 16, 2021).

[19] Liu L Feigin V Sacco RL Koroshetz WJ. Promoting global collaboration for brain health research. BMJ. (2020) 371:m3753. 10.1136/bmj.m3753 33036994 PMC7545087

[20] Lisko I Kulmala J Annetorp M Ngandu T Mangialasche F Kivipelto M. How can dementia and disability be prevented in older adults: where are we today and where are we going? J Intern Med. (2021) 289:807–30. 10.1111/joim.13227 33314384 PMC8248434

[21] National Assembly of the Republic of Slovenia. Resolution on the National Health Care Plan 2016–2025. Together for a Health Society. Off Gaz Repub Slov. (2016). p. 3407. Available online at: https://www.uradni-list.si/glasilo-uradni-list-rs/vsebina/2016-01-0999?sop=2016-01-0999 (accessed January 31, 2021).

[22] Ministry of Health of the Republic of Slovenia. Slovenia National Dementia Strategy 2016-2020. (2016). Available online at: https://www.zod-lj.si/images/Strategija_obvladovanja_demence.pdf (accessed January 31, 2021).

[23] National Institutes of Health. Estimates of Funding for Various Research, Condition, and Disease Categories (2020). Available online at: https://report.nih.gov/funding/categorical-spending#/ (accessed January 31, 2021).

[24] Illes J Moser MA McCormick JB Racine E Blakeslee S Caplan A. Neurotalk: improving the communication of neuroscience research. Nat Rev Neurosci. (2010) 11:61–9. 10.1038/nrn2773 19953102 PMC2818800

[25] Palma J-A Urrestarazu E Iriarte J. Sleep loss as risk factor for neurologic disorders: a review. Sleep Med. (2013) 14:229–36. 10.1016/j.sleep.2012.11.019 23352029

[26] Ju Y-ES Lucey BP Holtzman DM. Sleep and Alzheimer disease pathology—a bidirectional relationship. Nat Rev Neurol. (2014) 10:115–9. 10.1038/nrneurol.2013.269 24366271 PMC3979317

[27] Meruelo AD Castro N Cota CI Tapert SF. Cannabis and alcohol use, and the developing brain. Behav Brain Res. (2017) 325:44–50. 10.1016/j.bbr.2017.02.025 28223098 PMC5406224

[28] FutuNatura. Ginko Biloba. Available online at: https://www.futunatura.si/ginkgo-biloba-spomin (accessed January 28, 2021).

[29] Canter PH Ernst E. Ginkgo biloba is not a smart drug: an updated systematic review of randomised clinical trials testing the nootropic effects of G. biloba extracts in healthy people. Hum Psychopharmacol. (2007) 22:265–78. 10.1002/hup.843 17480002

[30] Demertzi A Liew C Ledoux D Bruno MA Sharpe M Laureys S. Dualism persists in the science of mind. Ann N Y Acad Sci. (2009) 1157:1–9. 10.1111/j.1749-6632.2008.04117.x 19351351

[31] Ohrnberger J Fichera E Sutton M. The relationship between physical and mental health: a mediation analysis. Soc Sci Med. (2017) 195:42–9. 10.1016/j.socscimed.2017.11.008 29132081

[32] Kolappa K Henderson C Kishore SP. No physical health without mental health : lessons unlearned? (2013) 91:3–3A. 10.2471/BLT.12.115063 23397342 PMC3537253

[33] Robson D Gray R. Serious mental illness and physical health problems: a discussion paper. Int J Nurs Stud. (2007) 44:457–66. 10.1016/j.ijnurstu.2006.07.013 17007859

[34] World Health Organisation. Global Recommendations on Physical Activity for Health. Available online at: https://www.who.int/dietphysicalactivity/physical-activity-recommendations-18-64years.pdf (accessed April 2, 2021).

[35] National Assembly of the Republic of Slovenia. Resolution on the national program on nutrition and physical activity for health 2015-2025. Off Gaz Repub Slov. (2015). Available online at: http://pisrs.si/Pis.web/pregledPredpisa?id=RESO101# (accessed January 31, 2021).

[36] AHA/ASA. The American Heart Association Diet and Lifestyle Recommendations. Available online at: https://www.heart.org/en/healthy-living/healthy-eating/eat-smart/nutrition-basics/aha-diet-and-lifestyle-recommendations (accessed April 2, 2021).

[37] AHA/ASA. American Heart Association Recommendations for Physical Activity in Adults and Kids. Available online at: https://www.heart.org/en/healthy-living/fitness/fitness-basics/aha-recs-for-physical-activity-in-adults (accessed April 2, 2021).

[38] World Health Organisation. Healthy Diet. Available online at: https://www.who.int/news-room/fact-sheets/detail/healthy-diet (accessed April 2, 2021).

[39] National Assembly of the Republic of Slovenia. Resolution on the national mental health program 2018-2028. Off Gaz Repub Slov. (2018). p. 3575. Available online at: https://www.uradni-list.si/glasilo-uradni-list-rs/vsebina/2018-01-1046/resolucija-o-nacionalnem-programu-dusevnega-zdravja-2018-2028-renpdz18-28 (accessed January 31, 2021).

[40] Wang Y McKee M Torbica A Stuckler D. Systematic literature review on the spread of health-related misinformation on social media. Soc Sci Med. (2019) 240:112552. 10.1016/j.socscimed.2019.112552 31561111 PMC7117034

[41] Swire-Thompson B Lazer D. Public health and online misinformation: challenges and recommendations. Annu Rev Public Health. (2020) 41:433–451. 10.1146/annurev-publhealth-040119-094127 31874069

[42] European Science Foundation. Plan S. (2018). Available online at: https://www.coalition-s.org/ (accessed January 31, 2021).

[43] Schiltz M. Science without publication paywalls: cOAlition S for the realisation of full and immediate Open Access. PLoS Biol. (2018) 16:e3000031. 10.1371/journal.pbio.3000031 30178783 PMC6122174

[44] Bouton ME. Why behavior change is difficult to sustain. Prev Med. (2014) 68:29–36. 10.1016/j.ypmed.2014.06.010 24937649 PMC4287360

[45] Kelly MP Barker M. Why is changing health-related behaviour so difficult? Public Health. (2016) 136:109–16. 10.1016/j.puhe.2016.03.030 27184821 PMC4931896

[46] Kwasnicka D Dombrowski SU White M Sniehotta F. Theoretical explanations for maintenance of behaviour change: a systematic review of behaviour theories. Health Psychol Rev. (2016) 10:277–96. 10.1080/17437199.2016.1151372 26854092 PMC4975085

[47] Fadhil A Matteotti C Armellin G Villafiorita A Betti D. CoachMe: a platform for promoting healthy lifestyle. In: Proceedings of the 18th International Conference on Human-Computer Interaction with Mobile Devices and Services Adjunct. New York, NY: ACM (2016). p. 1077–80.

[48] Cerar K Kondrič M Sindik J. The profiling of university of Ljubljana students according to their motives for exercise participation. Slov J Public Heal. (2017) 56:107–14. 10.1515/sjph-2017-0014 28289470 PMC5329774

[49] Kreuter MW Lukwago SN Bucholtz DC Clark EM Sanders-Thompson V. Achieving cultural appropriateness in health promotion programs: targeted and tailored approaches. Heal Educ Behav. (2003) 30:133–46. 10.1177/1090198102251021 12693519

[50] Kumar S Preetha G. Health promotion: an effective tool for global health. Indian J Community Med. (2012) 37:5. 10.4103/0970-0218.94009 22529532 PMC3326808

[51] Schwarz AF Huertas-Delgado FJ Cardon G DeSmet A. Design features associated with user engagement in digital games for healthy lifestyle promotion in youth: a systematic review of qualitative and quantitative studies. Games Health J. (2020) 9:150–63. 10.1089/g4h.2019.0058 31923363

[52] Mosa ASM Yoo I Sheets L. A systematic review of healthcare applications for smartphones. BMC Med Inform Decis Mak. (2012) 12:67. 10.1186/1472-6947-12-67 22781312 PMC3534499

[53] Higgins JP. Smartphone applications for patients' health and fitness. Am J Med. (2016) 129:11–9. 10.1016/j.amjmed.2015.05.038 26091764

[54] Golja P Robič T. The role of sports clubs in sports activity of students. Slov J Public Heal. (2014) 53:26–33. 10.2478/sjph-2014-0004

[55] Coles E Cheyne H Daniel B. Early years interventions to improve child health and well-being: what works, for whom and in what circumstances? Protocol for a realist review. Syst Rev. (2015) 4:79. 10.1186/s13643-015-0068-5 26047950 PMC4464136

[56] Lau RR Quadrel MJ Hartman KA. Development and change of young adults' preventive health beliefs and behavior: influence from parents and peers. J Health Soc Behav. (1990) 31:240. 10.2307/2136890 2133479

[57] Frech A. Advances in life course research healthy behavior trajectories between adolescence and young adulthood. Adv Life Course Res. (2012) 17:59–68. 10.1016/j.alcr.2012.01.003 22745923 PMC3381431

[58] Middleton LE Barnes DE Lui L-Y Yaffe K. Physical activity over the life course and its association with cognitive performance and impairment in old age. J Am Geriatr Soc. (2010) 58:1322–26. 10.1111/j.1532-5415.2010.02903.x 20609030 PMC3662219

[59] Lv J Yu C Guo Y Bian Z Yang L Chen Y. Adherence to healthy lifestyle and cardiovascular diseases in the Chinese population. J Am Coll Cardiol. (2017) 69:1116–25. 10.1016/j.jacc.2016.11.076 28254173 PMC6675601

[60] Emery CA Black AM Kolstad A Martinez G Nettel-Aguirre A Engebretsen L. What strategies can be used to effectively reduce the risk of concussion in sport? A systematic review. Br J Sports Med. (2017) 51:978–84. 10.1136/bjsports-2016-097452 28254746

[61] Brådvik L. Suicide risk and mental disorders. Int J Environ Res Public Health. (2018) 15:2028. 10.3390/ijerph15092028 PMC6165520 30227658

[62] Pelkonen M Marttunen M. Child and adolescent suicide. Pediatr Drugs. (2003) 5:243–65. 10.2165/00128072-200305040-00004 12662120

[63] World Health Organisation. Suicide Data. (2019). Available online at: https://www.who.int/teams/mental-health-and-substance-use/suicide-data (accessed January 21, 2021).

[64] Almberg B Grafstro M. Caring for a demented elderly person — burden and burnout among caregiving relatives. J Adv Nurs. (1997) 25:109–16. 10.1046/j.1365-2648.1997.1997025109.x 9004018

[65] Livingston G Sommerlad A Orgeta V Costafreda SG Huntley J Ames D. Dementia prevention, intervention, and care: 2020 report of the Lancet commission. Lancet. (2020) 390:2673–734. 10.1016/S0140-6736(17)31363-6 32738937 PMC7392084

[66] Chiuve SE Rexrode KM Spiegelman D Logroscino G Manson JE Rimm EB. Primary prevention of stroke by healthy lifestyle. Circulation. (2008) 118:947–54. 10.1161/CIRCULATIONAHA.108.781062 18697819 PMC2730914

[67] Jacka FN Mykletun A Berk M. Moving towards a population health approach to the primary prevention of common mental disorders. BMC Med. (2012) 10:149. 10.1186/1741-7015-10-149 23186355 PMC3534562

[68] Dias A Ferri C Graham N Ineichen B Prince M Uwakwe R. Neurological disorders: a public health approach. In: Neurological Disorders: Public Health Challenges. Geneva: WHO Press. p. 42–55. Available online at: https://www.who.int/mental_health/neurology/chapter_3_b_neuro_disorders_public_h_challenges.pdf?ua=1.

[69] Smith BJ Ali S Quach H. Public knowledge and beliefs about dementia risk reduction: a national survey of Australians. BMC Public Health. (2014) 14:661. 10.1186/1471-2458-14-661 24972448 PMC4226999

[70] Hosking DE Sargent-Cox KA Anstey KJ. An Australian survey of cognitive health beliefs, intentions, and behaviours through the adult life course. Prev Med Rep. (2015) 2:498–504. 10.1016/j.pmedr.2015.06.008 26844109 PMC4721299

[71] Centers for disease control and prevention. What is a Healthy Brain? New Research Explores Perceptions of Cognitive Health Among Diverse Older Adults. (2009). Available online at: https://www.cdc.gov/aging/pdf/perceptions_of_cog_hlth_factsheet.pdf (accessed May 29, 2021).

[72] Budin-Ljøsne I Friedman BB Suri S Solé-Padullés C Düzel S Drevon CA. The global brain health survey: development of a multi-language survey of public views on brain health. Front Public Heal. (2020) 8:387. 10.3389/fpubh.2020.00387 32923418 PMC7456866

[73] Statistical office of the Republic of Slovenia. Slovene Statistical Regions and Municipalities in Numbers. Available online at: https://www.stat.si/obcine/sl (accessed January 21, 2021).

[74] Statistical office of the Republic of Slovenia. Population, Slovenia, 1 January 2019. Available online at: https://www.stat.si/statweb/news/index/8062 (accessed January 21, 2021).

[75] Statistical office of the Republic of Slovenia. Population Aged 15 Years or More by Activity Status, Sex and Education, Slovenia, Annually. Available online at: https://pxweb.stat.si/SiStatData/pxweb/sl/Data/Data/05G3004S.px/ (accessed January 31, 2021).

[76] Statistical office of the Republic of Slovenia. Participants in Formal Education by Type of Program and Sex, Slovenia, Annually. Available online at: https://pxweb.stat.si/SiStatData/pxweb/sl/Data/-/0951305S.px (accessed January 31, 2021).

