# Supplementary material for: Lay Public View of Neuroscience and Science-Based Brain Health Recommendations in Slovenia
Source: Front Public Health. 2021 Jul 1;9:690421. doi: 10.3389/fpubh.2021.690421 (PMC8281117; doi:10.3389/fpubh.2021.690421)
Supplement: Supplementary file 1 [file Table_1.DOCX]

**Appendix: English translation of the survey**

1. Gender

- Male
- Female
- Other

1. State your age (in years): _____________
2. In which region do you live?

- Central Slovenia
- Pomurje
- Podravje
- Koroška
- Savinjska
- Zasavje
- Southeastern Slovenija
- Gorenjska
- Goriška
- Adriatic Coast and Carst
- Posavje
- Primorska and Notranjska

1. What is your accomplished level of education?

- Unfinished basic school (I)
- Basic school leaving certificate (II)
- Short upper secondary vocational education (III)
- Upper secondary vocational education (IV)
- Upper secondary technical education; upper secondary general education; national vocational qualification; foreman’s examination certificate (V)
- Short cycle higher vocational diploma (VI)
- Professional bachelor’s education; academic bachelor’s education; pre-bologna professional higher education; specialization diploma following old short cycle higher education (VII)
- Master’s degree; diploma of academic higher education; specialization diploma following professional higher education; higher education (VIII)
- Specialization diploma following academic higher education; specialization following professional higher education; pre-Bologna Research Master's Degree (IX)
- Doctorate (X)

1. What applies to you? (Multiple answers possible)

- I am employed
- I am unemployed
- I am engaged in educational process
- I am retired
- Other: _____________

1. Which of the following applies to you? (Multiple answers possible)

- I have/am in the process of formal education which includes systematic learning about structure and functioning of the brain
- I rely on my knowledge about structure and functioning of the brain at my workplace
- I actively educate others about brain-related subjects
- None of the above applies to me

1. Have you ever been diagnosed with neurological or psychiatric disease (e.g. brain stroke, brain tumor, dementia, schizophrenia, epilepsy, Parkinson’s disease, migraine, sleep disorders, depression, addiction, anxiety etc.)?

- Yes (please state the diagnosis: _____________)
- No

1. Has any of your relatives (family member, close friend, partner) ever been diagnosed with neurological or psychiatric disease (e.g. brain stroke, brain tumor, dementia, schizophrenia, epilepsy, Parkinson’s disease, migraine, sleep disorders, depression, addiction, anxiety etc.)?

- Yes (please state the diagnosis: _____________)
- No

1. How important is to you that your brain is healthy and well-functioning?

- Health and good functioning of the brain are not important to me at all
- Many things are more important to me than health and good functioning of the brain
- Health and good functioning of the brain are equally important as other things in life
- Health and good functioning of the brain are important and one must take care of it as much as possible
- Health and good functioning of the brain are one of the most important things in life

1. How would you evaluate your knowledge of the brain in regard to your needs?

- I have no/almost no knowledge of the brain
- I have some knowledge of the brain
- My knowledge is relatively sufficient
- My knowledge is good enough to cover my needs
- I have excellent knowledge of the brain

1. How important for brain health do you find the following practices?

|  | Not at all | A little | Medium | | Quite a lot | | Very much | |
| --- | --- | --- | --- | --- | --- | --- | --- | --- |
| Eating healthy | 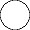 | 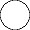 | 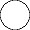 | 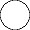 | | 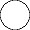 | |  |
| Exercise | 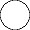 | 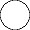 | 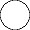 | 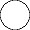 | | 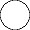 | |  |
| Enough sleep | 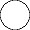 | 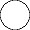 | 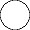 | 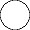 | | 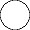 | |  |
| Avoiding drugs and alcohol | 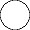 | 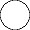 | 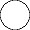 | 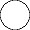 | | 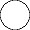 | |  |
| Avoiding injury (wearing a helmet, avoiding contact sports) | 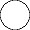 | 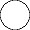 | 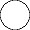 | 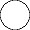 | | 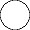 | |  |
| Relaxation and rest during work | 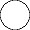 | 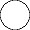 | 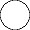 | 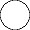 | | 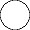 | |  |
| Maintaining social contacts (e.g. socializing with friends) | 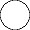 | 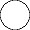 | 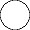 | 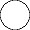 | | 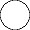 | |  |
| Acquiring new knowledge and skills (language, instruments, learning new things) | 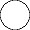 | 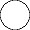 | 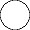 | 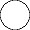 | | 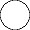 | |  |
| Mental challenges and cognitive training | 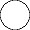 | 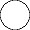 | 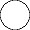 | 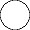 | | 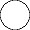 | |  |
| Using supplements that are considered to improve mental abilities | 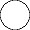 | 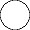 | 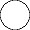 | 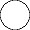 | | 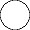 | |  |

1. To what extent do you comply with recommendations for brain health?

|  | Never | Rarely | Sometimes | | Often | | Every day | |
| --- | --- | --- | --- | --- | --- | --- | --- | --- |
| Eating healthy | 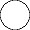 | 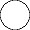 | 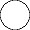 | 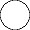 | | 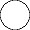 | |  |
| Exercise | 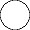 | 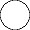 | 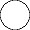 | 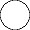 | | 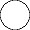 | |  |
| Enough sleep | 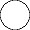 | 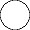 | 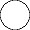 | 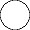 | | 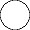 | |  |
| Avoiding drugs and alcohol | 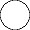 | 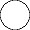 | 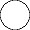 | 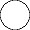 | | 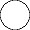 | |  |
| Avoiding injury (wearing a helmet, avoiding contact sports) | 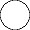 | 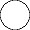 | 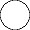 | 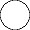 | | 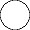 | |  |
| Relaxation and rest during work | 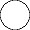 | 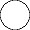 | 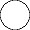 | 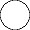 | | 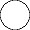 | |  |
| Maintaining social contacts (e.g. socializing with friends) | 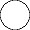 | 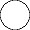 | 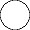 | 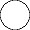 | | 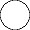 | |  |
| Acquiring new knowledge and skills (language, instruments, learning new things) | 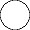 | 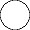 | 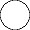 | 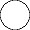 | | 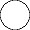 | |  |
| Mental challenges and cognitive training | 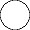 | 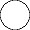 | 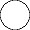 | 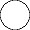 | | 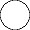 | |  |
| Using supplements that are considered to improve mental abilities | 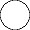 | 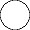 | 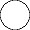 | 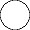 | | 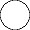 | |  |

1. Why don't you engage in the following activities as much as you want/at all? (Multiple answers possible)

- I engage in all the activities as much as I want
- I do not have enough time
- I do not have enough information
- I do not think these activities are important enough to spend time or money on them
- Most people I know do not engage in these activities
- I will engage in these activities if I get sick
- I am too young/old to engage in these activities
- I do not have the opportunity due to finances, transportation issues or other life circumstances
- Other: _____________

1. For which activities do you lack time? (Multiple answers possible)

- Eating healthy
- Exercise
- Enough sleep
- Relaxation and rest during work
- Maintaining social contacts (e.g. socializing with friends)
- Acquiring new knowledge and skills (language, instruments, learning new things)
- Mental challenges and cognitive training
- Other: _____________

1. For which activities do you lack information? (Multiple answers possible)

- Eating healthy
- Exercise
- Enough sleep
- Relaxation and rest during work
- Maintaining social contacts (e.g. socializing with friends)
- Acquiring new knowledge and skills (language, instruments, learning new things)
- Mental challenges and cognitive training
- Using supplements that are considered to improve mental abilities
- Other: _____________

1. Do you believe that the probability of the onset of any of the diseases and disorders listed below could be decreased by a healthy lifestyle and taking care of the brain? (Multiple answers possible)

- We have no influence on the onset of these diseases and disorders
- Dementia
- Eating disorders (e.g. anorexia, bulimia)
- Mood disorders (e.g. depression, bipolar disorder)
- Brain stroke
- Anxiety and specific phobias
- Sleep disorders
- Headache
- Addiction
- Parkinson’s disease
- Multiple sclerosis

1. How often do you actively seek information about the brain?

- Often
- Occasionally
- Never

1. Which sources do you most often use to gain information about the brain? (Maximum three answers possible)

- Newspapers/magazines
- Popular explanation books
- Scientific articles/books
- Lectures/workshops
- TV
- Radio
- Professionals (e.g. doctors, psychologists)
- Conversation with others (e.g. friends)
- Internet (Which websites? _____________)
- Other: _____________

1. What is your preferred manner of acquiring new knowledge about brain, brain disease and preventive practices? Rank the listed options from the most (1) to least preferable (9).

- Formal education
- Popular explanation books/articles
- Scientific books/articles
- Lectures by experts
- Workshops
- Listening to experience of people with brain disorders
- TV/radio shows
- Official websites maintained by experts
- Internet regardless of the source

1. Have you met any obstacles in searching for information about the brain? (Multiple answers possible)

- I have not encountered any obstacles
- There is not enough information in Slovene
- I have no time and/or motivation to search for information
- I do not know which sources to trust
- Content is not presented in an interesting way
- Payable and expensive access to content
- Other: _____________
